# Cannabis, schizophrenia genetic risk, and psychotic experiences: a cross-sectional study of 109,308 participants from the UK Biobank

**DOI:** 10.1038/s41398-021-01330-w

**Published:** 2021-04-09

**Authors:** Michael Wainberg, Grace R. Jacobs, Marta di Forti, Shreejoy J. Tripathy

**Affiliations:** 1 Centre for Addiction and Mental Health, Toronto, ON Canada; 2 Institute of Medical Sciences, University of Toronto, Toronto, ON Canada; 3 Institute of Psychiatry, Psychology and Neuroscience, King’s College London, London, UK; 4 South London and Maudsley NHS Mental Health Foundation Trust, London, UK; 5 Department of Psychiatry, University of Toronto, Toronto, ON Canada; 6 Department of Physiology, University of Toronto, Toronto, ON Canada

**Keywords:** Genetics, Schizophrenia

## Abstract

Cannabis is known to produce acute, transient psychotic-like experiences. However, it is unclear whether cannabis disproportionately increases the risk of specific types of psychotic experiences and whether genetic predisposition influences the relationship between cannabis use and psychotic experiences. In this cross-sectional study of 109,308 UK Biobank participants, we examined how schizophrenia polygenic risk modulates the association between self-reported cannabis use and four types of self-reported psychotic experiences (auditory hallucinations, visual hallucinations, persecutory delusions, and delusions of reference). Cohort-wide, we found a strong, dose-dependent relationship between cannabis use and all four types of psychotic experiences, especially persecutory delusions. Cannabis users’ psychotic experiences tended to be earlier-onset and cause greater distress than non-users’, but were not more likely to lead to help-seeking. Participants with high schizophrenia polygenic risk scores showed stronger associations between cannabis use and auditory hallucinations, visual hallucinations, and delusions of reference, as well as psychotic experiences overall. For instance, cannabis ever-use was associated with 67% greater adjusted odds of delusions of reference among individuals in the top fifth of polygenic risk, but only 7% greater adjusted odds among the bottom fifth. Our results suggest that cannabis use is a predictive risk factor for psychotic experiences, including early-onset and distressing experiences. Individuals genetically predisposed to schizophrenia may be especially vulnerable to psychotic experiences as a result of using cannabis, supporting a long-postulated hypothesis. This study exemplifies the utility of population-scale biobanks for elucidating gene-by-environment interactions relating substance use to neuropsychiatric outcomes and points to the translational potential of using polygenic risk scores to inform personalized harm reduction interventions.

## Introduction

Substantial epidemiological evidence associates cannabis use with psychosis^1^ and accelerated age of onset of psychosis^2,3^, although the causality of these relationships have long been debated^1,4^. What is incontrovertible is that cannabis can induce acute psychotic-like experiences in healthy individuals, although these are generally mild and transient^5^. The association of cannabis use with psychotic-like experiences appears largely genetically mediated, albeit with some environmental contribution^6–8^.

Multinational population-based surveys suggest self-reported psychotic experiences—whether caused by cannabis or not—are several times more common than diagnosed psychotic disorders^9^. Despite most not being sufficiently severe to merit a diagnosis, psychotic experiences nonetheless predict poor outcomes including psychotic disorders^10–12^, supporting their clinical relevance. A recent meta-analysis found a dose-dependent relationship between cannabis use and a variety of psychosis-related outcomes, including self-reported psychotic experiences^13^.

Cannabis use may have different relationships with different types of psychotic experiences. Although cannabis was historically classified as a hallucinogen based on the acute perceptual changes it tends to induce^14^, this categorization is considered controversial^15^ and case reports of bona fide cannabis-induced hallucinations are rare^15,16^. On the other hand, delusions resulting from cannabis use appear much more common: cannabis readily induced delusional thinking in multiple instances within a single randomized control trial^5^ and cannabis use has been linked to persecutory ideation^17^ and paranoia^18^. Although these observations relate primarily to the acute effects of cannabis intoxication, the same dichotomy between hallucinations and delusions may also apply in the longer term. For instance, each year of regular marijuana use among adolescent boys was associated with an odds ratio of 1.92 for hallucinations, but an even greater odds ratio of 2.33 for paranoia^19^.

Genetic studies have implicated the endocannabinoid system in psychotic experiences: the strongest association with distressing psychotic experiences in a recent genome-wide association study (GWAS) was the *CNR2* (cannabinoid receptor 2) locus^10^. Genetics may also modulate susceptibility to cannabis-related harm: the association between cannabis use and decreased cortical thickness, a risk factor for psychotic experiences, among early adolescent males is strongest among those with the highest polygenic risk for schizophrenia^20^. However, to our knowledge, no study has directly tested the hypothesis^21,22^ that genetic predisposition to schizophrenia makes cannabis users especially liable to psychotic experiences.

Here we sought to investigate the association between cannabis use and self-reported psychotic experiences in a population-scale cohort, the UK Biobank, with several questions in mind. First, whether cannabis use is differentially associated with different categories of self-reported psychotic experiences. The UK Biobank asks about four types—auditory hallucinations, visual hallucinations, persecutory delusions, and delusions of reference—and we set out to characterize cannabis’s associations with each one, with appropriate correction for multiple testing. Second, whether cannabis users’ self-reported psychotic experiences are different from non-users’ in terms of age of onset^2,3^, distress, and likelihood of help-seeking. Third, whether the association of cannabis use with any of the four types of self-reported psychotic experiences is more pronounced among individuals genetically predisposed to schizophrenia, once again rigorously correcting for multiple testing.

## Methods

### Participants

Participants were included from the UK Biobank, a prospective cohort study with genetics and deep phenotyping on ~500,000 British individuals, aged 40–69 years at recruitment. In total, 157,348 participants completed an online Mental Health Questionnaire^23^, of which 109,308 participants (61,047 female and 48,261 male) of unrelated White British ancestry (defined using the same criteria as a previous study^24^) met the inclusion criteria. Specifically, these participants answered questions on both cannabis use and psychotic experiences, and lacked a diagnosis of any psychotic disorder (ICD-10 codes F20–F29) according to linked inpatient, primary care, or death records (e.g., according to “Source of report of F20 (schizophrenia)”, Data-Field 130875). No individuals had an ICD code for F19.15 or F19.95 (drug-induced psychosis); thus, this was not used as a criterion for exclusion. This cohort formed the basis of our analyses.

### Definitions of self-reported cannabis use and psychotic experiences

Self-reported cannabis use was defined by the “Ever taken cannabis” question (Data-Field #20453; Table 1). Use frequency was defined by the “Maximum frequency of taking cannabis” question (#20454: ever-use = 1, *N* = 14,642; monthly use = 2, *N* = 2671; weekly use = 3, *N* = 3582; daily use = 4, *N* = 1403); individuals answering no to “Ever taken cannabis” were not asked this question and assigned a value of 0 (*N* = 87,010).

**Table 1:** Cannabis use and psychotic experience questions from the UK Biobank Mental Health Questionnaire.

**Categories of self-reported psychotic experiences**	
Auditory hallucinations	Did you ever hear things that other people said did not exist, like strange voices coming from inside your head talking to you or about you, or voices coming out of the air when there was no one around?
Visual hallucinations	Did you ever see something that wasn’t really there that other people could not see?
Persecutory delusions	Did you ever believe that there was an unjust plot going on to harm you or to have people follow you, and which your family and friends did not believe existed?
Delusions of reference	Did you ever believe that a strange force was trying to communicate directly with you by sending special signs or signals that you could understand but that no one else could understand (e.g., through the radio or television)?
**Qualities of self-reported psychotic experiences**	
Age of onset	How old were you (approximately) when you first had one of these experiences?
Distress	How distressing did you find having any of these experiences?
Help-seeking	Did you ever talk to a doctor, counselor, psychiatrist, or other health professionals about any of these experiences?
**Self-reported cannabis use**	
Cannabis ever-use	Have you taken cannabis (marijuana, grass, hash, ganja, blow, draw, skunk, weed, spliff, dope), even if it was a long time ago?
Cannabis use frequency	Considering when you were taking cannabis most regularly, how often did you take it?

Self-reported psychotic experiences were defined as auditory (#20463) or visual (#20471) hallucinations, or delusions of persecution (#20468) or reference (#20474), as in previous studies of the UK Biobank^10,25^. The number of individuals of the 109,308 with non-missing data for each individual psychotic experience ranged from 108,174 for visual hallucinations to 109,104 for persecutory delusions. Self-reported age of onset was ascertained from the “Age when first had unusual or psychotic experience” question (#20461), distress from “Distress caused by unusual or psychotic experiences” (#20462), and help-seeking from “Ever talked to a health professional about unusual or psychotic experiences” (#20477).

### Schizophrenia polygenic risk score

A polygenic risk score (PRS) for schizophrenia was computed for each participant, based on a recent GWAS^26^ (walters.psycm.cf.ac.uk/clozuk_pgc2.meta.sumstats.txt.gz) from an independent cohort. First, samples were subset to genetically defined White British (“Genetic ethnic grouping” [Data-Field #22006] is 1) without sex chromosome aneuploidy (missing value for “Sex chromosome aneuploidy” [#22019]), who were used to compute genotype principal components (non-missing value for “Used in genetic principal components” [#22020]). Second, variants from the UK Biobank’s imputed genotypes were subset to non-duplicate, autosomal single-nucleotide polymorphisms with call rate > 95%, Hardy–Weinberg equilibrium *p*-value > 1 × 10^−10^, allele frequency > 0.1%, and imputation info score > 0.8. Third, GWAS summary statistics were harmonized with the UK Biobank with respect to reference/alternate allele and strand; ambiguous variants (A/T, C/G, G/C, and T/A) and variants missing from UK Biobank were excluded. Fourth, variants were filtered to *p* < 0.05. This *p* < 0.05 threshold led to a better prediction of schizophrenia across the unrelated White British individuals (area under the curve [AUC] = 0.677) than stricter *p*-value thresholds of 0.005 (AUC = 0.648), 0.0005 (AUC = 0.612), or 0.00005 (AUC = 0.586). (The AUC, also known as the area under the receiver operating characteristic curve or concordance statistic, is the fraction of the time that the PRS would rank a randomly chosen case higher than a randomly chosen control.) Fifth, linkage disequilibrium pruning to *r*^2^ < 0.5 was performed using a 500 kb sliding window. The effect sizes (log odds ratios) of the remaining variants constituted the weights of the PRS. The PRS was scored on each individual in the cohort by summing, across the variants in the PRS, the variant’s weight times the individual’s number of effect alleles of that variant; missing genotypes were mean-imputed.

### Statistical analysis

Raw prevalences of each self-reported psychotic experience were tabulated among ever- and never-users. Covariate-corrected adjusted odds ratios (AORs) and associated 95% confidence intervals were also calculated, via logistic regression of each psychotic experience on cannabis ever-use (coded as binary variables) and covariates, using the *glm* function in R. As a sensitivity analysis, cannabis use frequency (coded as never-use = 0, ever-use = 1, monthly use = 2, weekly use = 3, daily use = 4) was used in the logistic regression instead of cannabis ever-use. Covariates consisted of birth year (Data-Field #34), sex (#31), educational qualifications (#6138), pre-tax household income (#738), employment status (#6142), Townsend deprivation index (#189), Index of Multiple Deprivation (#26410, #26426, and #26427), smoking status (#20116), alcohol intake frequency (#1558), UK Biobank assessment center (#54), and the top ten genotype principal components (#22009). Categorical covariates were coded as indicator variables.

*P*-values for additive interactions with schizophrenia genetic risk were calculated using the *glm* function by performing linear regression of psychotic experiences on three variables (plus covariates)—the exposure (either cannabis ever-use or cannabis use frequency), the schizophrenia PRS, and the product of the two (i.e., the interaction term)—then performing a *χ*^2^-test using the *anova* function in R, to compare this model to a simpler two-variable model (plus covariates) lacking the interaction term. To properly control for confounding^27^, covariate-by-exposure and covariate-by-PRS interaction terms were also included as covariates in both models. Multiple testing correction was performed using Benjamini–Hochberg correction at a standard false discovery rate (FDR) of 10%.

*P*-values for the difference *D* between pairs of logistic regression coefficients (log odds ratios) were calculated by computing a SE for the difference as the root sum of squares of the coefficients’ SEs, dividing *D* by this SE to yield a *Z*-score, then inverse-normal transforming.

## Results

### Self-reported psychotic experiences strongly correlate with cannabis use frequency

When considering the cohort as a whole, we found a strong and consistent relationship between self-reported cannabis use frequency and all types of self-reported psychotic experiences (Table 2). Although 4.1% of cannabis never-users reported one of the four types of experiences surveyed (auditory hallucinations, visual hallucinations, persecutory delusions, or delusions of reference), this rose to 7.0% among ever-users (AOR = 1.54 [1.43, 1.65]) and rose further to 8.4% among those reporting ever using cannabis at least monthly (AOR = 1.69 [1.54, 1.87]), 8.8% among ever-weekly users (AOR = 1.69 [1.51, 1.89]), and 9.6% among ever-daily users (AOR = 1.79 [1.52, 2.20]). Defining never-use as 0 “risk units”, ever-use as 1 “risk unit”, and so forth up to 4 “risk units” for daily use, we found that the odds of any of the four psychotic experiences increased by 20% per risk unit (AOR = 1.20 [1.16, 1.24]).

**Table 2:** Self-reported psychotic experiences are strongly associated with cannabis use frequency.

	Prevalence of self-reported psychotic experiences in the UK Biobank, stratified by self-reported cannabis use frequency					AOR per risk unit
	Never (0 risk units)	Ever (1 risk unit)	Monthly (2 risk units)	Weekly (3 risk units)	Daily (4 risk units)	
Any psychotic experience	4.1%	7.0% AOR = **1.54** [1.43, 1.65]	8.4% AOR = **1.69** [1.54, 1.87]	8.8% AOR = **1.69** [1.51, 1.89]	9.6% AOR = **1.79** [1.52, 2.20]	AOR = **1.20** [1.16, 1.24]
Auditory hallucinations	1.3%	2.7% AOR = **1.57** [1.40, 1.77]	3.4% AOR = **1.84** [1.57, 2.16]	3.6% AOR = **1.85** [1.55, 2.21]	3.6% AOR = **1.79** [1.38, 2.33]	AOR = **1.21** [1.15, 1.27]
Visual hallucinations	2.8%	4.6% AOR = **1.58** [1.45, 1.73]	5.4% AOR = **1.69** [1.50, 1.91]	5.5% AOR = **1.66** [1.45, 1.91]	6.1% AOR = **1.76** [1.44, 2.15]	AOR = **1.21** [1.16, 1.26]
Persecutory delusions	0.6%	1.3% AOR = **1.59** [1.34, 1.89]	1.8% AOR = **1.95** [1.56, 2.44]	2.2% AOR = **2.14** [1.68, 2.74]	2.6% AOR = **2.44** [1.75, 3.40]	AOR = **1.24** [1.15, 1.33]
Delusions of reference	0.6%	0.9% AOR = **1.39** [1.15, 1.68]	1.2% AOR = **1.57** [1.21, 2.03]	1.3% AOR = **1.65** [1.24, 2.19]	1.4% AOR = **1.67** [1.10, 2.53]	AOR = **1.18** [1.08, 1.28]

Square brackets denote 95% confidence intervals. It is noteworthy that AORs are adjusted for covariates, while percentages are not.

The bold values are purely for visual emphasis.

A sensitivity analysis stratifying by sex (Table 3) indicated that the association of cannabis ever-use with psychotic experiences was significantly stronger among females than among males (AOR = 1.59 vs. 1.44). Two particular types of psychotic experiences, auditory hallucinations (AOR = 1.69 vs. 1.40) and delusions of reference (AOR = 1.67 vs. 1.20), also had significantly stronger associations among females.

**Table 3:** Sex differences in associations between psychotic experiences and cannabis ever-use.

	Prevalence of self-reported psychotic experiences (ever- vs. never-users)				
	Any psychotic experience	Auditory hallucinations	Visual hallucinations	Persecutory delusions	Delusions of reference
Female	8.0% vs. 4.5% AOR = **1.59** [1.45, 1.75]	3.0% vs. 1.5% AOR = **1.69** [1.45, 1.97]	5.5% vs. 3.1% AOR = **1.64** [1.47, 1.84]	1.0% vs. 0.5% AOR = **1.58** [1.21, 2.08]	0.9% vs. 0.5% AOR = **1.67** [1.28, 2.19]
Male	6.1% vs. 3.7% AOR = **1.44** [1.30, 1.61]	2.3% vs. 1.2% AOR = **1.40** [1.17, 1.67]	3.6% vs. 2.2% AOR = **1.51** [1.32, 1.74]	1.3% vs. 0.6% AOR = **1.59** [1.25, 2.02]	0.8% vs. 0.5% AOR = **1.20** [0.90, 1.60]
Difference	FDR = **6%** (*p* = 0.04)	FDR = **5%** (*p* = 0.01)	FDR = **20%** (*p* = 0.16)	FDR = **97%** (*p* = 0.97)	FDR = **5%** (*p* = 0.02)

FDRs are derived from Benjamini–Hochberg correction for five tests.

*FDR* false discovery rate.

The bold values are purely for visual emphasis.

### Cannabis use is particularly associated with persecutory delusions

Considering each of the four types of psychotic experiences individually (Table 2), we again found strong associations with cannabis ever-use (AOR = 1.39–1.59), and even stronger ones with monthly (AOR = 1.57–1.95), weekly (AOR = 1.65–2.14), and daily (AOR = 1.67–2.44) use. Persecutory delusions were especially strongly correlated (AOR = 2.44 [1.96, 3.64] for daily users; AOR = 1.24 [1.15, 1.33] per risk unit). Thus, when considering the cohort as a whole, we found a strong, dose-dependent relationship between cannabis use and all four types of psychotic experiences, particularly persecutory delusions.

### Cannabis ever-users report earlier-onset and more distressing experiences than never-users

Cannabis users were also especially likely to report early-onset (<18 years old) psychotic experiences (Table 4). Cannabis ever-users reported adult-onset psychotic experiences at greater rates than never-users (AOR = 1.52 [1.38, 1.66]), but even greater rates of early-onset experiences (AOR = 1.90 [1.64, 2.20]). (When calculating the association with adult-onset psychotic experiences, individuals with early-onset experiences were excluded, and vice versa.) Thus, although cannabis use was associated with both adult- and early-onset psychotic experiences, its association with early-onset experiences was significantly more pronounced (*p* = 1 × 10^−5^, FDR = 0.5%). Ever-users also reported disproportionately greater rates of distressing psychotic experiences (AOR = 1.62 [1.45, 1.81]) compared to non-distressing ones (AOR = 1.50 [1.37, 1.64]), but no greater rates of psychotic experiences leading to help-seeking (AOR = 1.45 [1.25, 1.70]) compared to ones not leading to help-seeking (AOR = 1.55 [1.43, 1.68]). Thus, ever-users’ psychotic experiences tended to be earlier-onset and more distressing than never-users’, but no more likely to lead to help-seeking.

**Table 4:** Cannabis ever-users report earlier-onset and more distressing psychotic experiences than never-users.

	Prevalence of self-reported psychotic experiences with particular qualities (ever- vs. never-users)		
	Early-onset (<18 years old)	Distressing	Associated with help-seeking
Had a psychotic experience that was ________	1.9% vs. 0.8% AOR = **1.90** [1.64, 2.20]	3.0% vs. 1.5% AOR = **1.62** [1.45, 1.81]	1.6% vs. 0.8% AOR = **1.45** [1.25, 1.70]
Had a psychotic experience that was not ________	4.1% vs. 2.5% AOR = **1.52** [1.38, 1.66]	4.1% vs. 2.6% AOR = **1.50** [1.37, 1.64]	5.6% vs. 3.3% AOR = **1.55** [1.43, 1.68]
Difference	FDR = **0.5%** (*p* = 1 × 10^−5^)	FDR = **9%** (*p* = 0.1)	FDR = **81%** (*p* = 0.3)

FDRs are derived from Benjamini–Hochberg correction for three tests; square brackets denote 95% confidence intervals.

*FDR* false discovery rate.

The bold values are purely for visual emphasis.

### Schizophrenia polygenic risk modulates the association of cannabis use with psychotic experiences

Finally, we considered whether a PRS for schizophrenia modulated the strengths of association between cannabis use and self-reported psychotic experiences (Table 5). We found that schizophrenia polygenic risk significantly interacted with cannabis ever-use to predict rates of auditory hallucinations (*p* = 0.02, FDR = 9%), delusions of reference (*p* = 0.04, FDR = 9%), and psychotic experiences overall (*p* = 0.05, FDR = 9%). Cannabis use frequency was better powered to detect interactions with schizophrenia polygenic risk, with significant results not only for auditory hallucinations (*p* = 0.01, FDR = 2%), delusions of reference (*p* = 0.0007, FDR = 0.4%), and psychotic experiences overall (*p* = 0.01, FDR = 2%), but also for visual hallucinations (*p* = 0.06, FDR = 7%).

**Table 5:** Schizophrenia PRS modulates the association of cannabis use with self-reported psychotic experiences.

	Prevalence of self-reported psychotic experiences (ever- vs. never-users), stratified by schizophrenia PRS					PRS-by-ever-use interaction	PRS-by-frequency interaction
	0–20th Percentile SCZ PRS	20–40th Percentile SCZ PRS	40–60th Percentile SCZ PRS	60–80th Percentile SCZ PRS	80–100th Percentile SCZ PRS		
Any psychotic experience	5.8 vs. 3.6% AOR = **1.39** [1.16, 1.65]	6.7 vs. 4.0% AOR = **1.48** [1.26, 1.74]	7.0 vs. 4.2% AOR = **1.52** [1.30, 1.78]	7.5 vs. 4.3% AOR = **1.64** [1.40, 1.91]	8.2 vs. 4.7% AOR = **1.58** [1.36, 1.83]	FDR = **9%** (*p* = 0.02)	FDR = **2%** (*p* = 0.01)
Auditory hallucinations	2.2 vs. 1.2% AOR = **1.36** [1.02, 1.82]	2.4 vs. 1.4% AOR = **1.32** [1.01, 1.73]	2.7 vs. 1.5% AOR = **1.54** [1.20, 1.98]	2.8 vs. 1.3% AOR = **1.91** [1.47, 2.48]	3.2 vs. 1.5% AOR = **1.73** [1.36, 2.22]	FDR = **9%** (*p* = 0.04)	FDR = **2%** (*p* = 0.01)
Visual hallucinations	3.7 vs. 2.4% AOR = **1.46** [1.18, 1.81]	4.6 vs. 2.7% AOR = **1.63** [1.34, 1.98]	4.5 vs. 2.7% AOR = **1.52** [1.25, 1.85]	4.9 vs. 3.0% AOR = **1.59** [1.31, 1.91]	5.2 vs. 3.0% AOR = **1.66** [1.38, 1.99]	FDR = **17%** (*p* = 0.1)	FDR = **7%** (*p* = 0.06)
Persecutory delusions	0.8 vs. 0.4% AOR = **1.60** [0.98, 2.60]	1.3 vs. 0.4% AOR = **2.30** [1.53, 3.45]	1.1 vs. 0.6% AOR = **1.65** [1.11, 2.44]	1.4 vs. 0.6% AOR = **1.39** [0.96, 2.00]	1.7 vs. 0.9% AOR = **1.36** [0.97, 1.89]	FDR = **99%** (*p* = 1)	FDR = **29%** (*p* = 0.3)
Delusions of reference	0.5 vs. 0.4% AOR = **1.07** [0.63, 1.82]	0.7 vs. 0.5% AOR = **1.29** [0.82, 2.05]	0.8 vs. 0.6% AOR = **1.07** [0.69, 1.67]	1.1 vs. 0.6% AOR = **1.54** [1.04, 2.28]	1.5 vs. 0.7% AOR = **1.68** [1.18, 2.38]	FDR = **9%** (*p* = 0.05)	FDR = **0.4%** (*p* = 0.0007)

FDRs for each type of interaction are derived from Benjamini–Hochberg correction for five tests. Square brackets denote 95% confidence intervals. It is noteworthy that our interaction tests (two right-most columns) treat polygenic risk as a continuous variable and do not rely on discretization into quintiles.

*FDR* false discovery rate.

The bold values are purely for visual emphasis.

To better interpret these interactions, we stratified individuals into quintiles (20-percentile bins) based on their schizophrenia polygenic risk and computed associations within each quintile between cannabis ever-use and each psychotic experience. We found that ever-use was associated with 1.58-fold [1.36, 1.58] greater adjusted odds of psychotic experiences among the one-fifth of individuals with the highest PRSs, compared to only 1.39-fold [1.16, 1.65] greater adjusted odds among the one-fifth with the lowest PRSs. This pattern also held true for auditory hallucinations (AOR = 1.73 [1.36, 2.22] among the top quintile vs. 1.36 [1.02, 1.82] among the bottom quintile) and visual hallucinations (AOR = 1.66 [1.38, 1.99] vs. 1.46 [1.18, 1.81]), and was particularly pronounced for delusions of reference, which had no significant association with cannabis use except among those in the top two-fifths of genetic risk. Thus, cannabis use was disproportionately highly correlated with psychotic experiences among individuals at high genetic risk of schizophrenia and less correlated among individuals at lower risk.

## Discussion

**Fig. 1:**
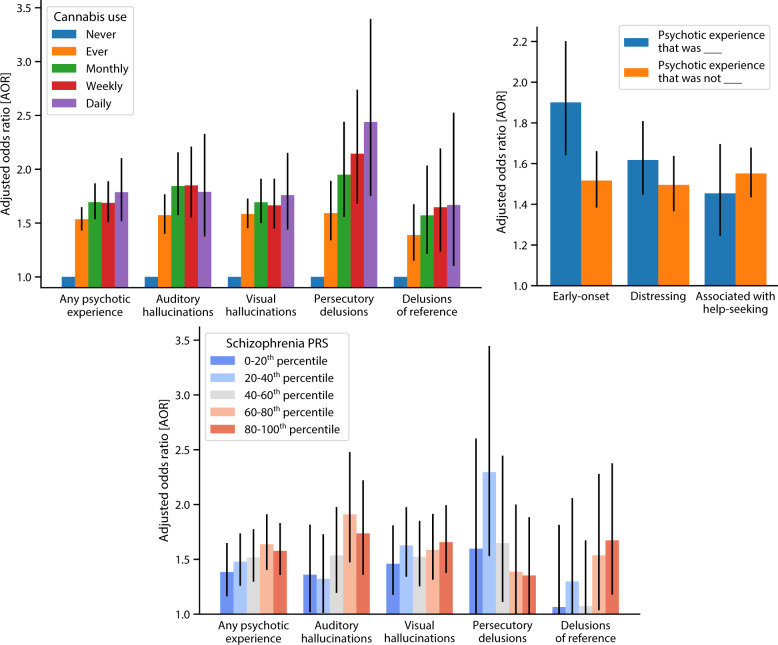
Summary of results. Cannabis use frequency correlates with all types of psychotic experiences (top left), cannabis ever-users report earlier-onset and more distressing psychotic experiences (top right), and schizophrenia PRS modulates the association of cannabis use with most types of psychotic experiences (bottom).

Our results suggest four main findings (Fig. 1). First, we confirm a strong, dose-dependent association between cannabis use and self-reported psychotic experiences, consistent across all four types of psychotic experiences surveyed—an important replication supported by the UK Biobank’s large sample size. Second, we find a particularly pronounced association of cannabis use with persecutory delusions. Third, we provide the first evidence of an association between cannabis use and earlier psychotic experience onset, extending prior studies showing such an association with diagnosed psychotic disorders^3,28^. Although cannabis ever-users report more distressing psychotic experiences than never-users, rates of help-seeking were similar in both groups. Fourth, we discover a strong modulatory effect of schizophrenia polygenic risk on cannabis’s association with multiple types of psychotic experiences. The difference in AORs between those in the bottom and top fifth of polygenic risk is strikingly large for delusions of reference (7% vs. 67%) and auditory hallucinations (36% vs. 74%), exemplifying the added value of population-scale biobanks for elucidating gene-by-environment interactions.

Notably, self-reported delusions of persecution and reference each have a special association with cannabis, but in different ways. Persecutory delusions have the strongest association with cannabis use frequency of all psychotic experiences surveyed, whereas delusions of reference are the sole type of delusion to interact with schizophrenia polygenic risk vis-a-vis cannabis use. It has been suggested that although both types of delusions involve frontostriatal prediction errors^29^, some brain regions involved may be specific to one type or the other. Paranoid delusions may specifically involve regions involved in theory of mind, in particular the right temporoparietal junction and right posterior superior temporal sulcus, as well as the amygdala, which is involved in paranoia-associated fear and hypervigilance, whereas delusions of reference may instead involve the associative striatum and nucleus basalis^30^. Cannabis is known to interact with the amygdala^31^, associative striatum^32^, and nucleus basalis^33^, and it is conceivable that cannabis’s interactions with delusions-of-reference-specific regions may be more modulated by schizophrenia-associated genetic factors than its interactions with persecutory-delusions-specific regions.

Our findings should be evaluated in the context of their limitations. In particular, it remains unclear to what extent cannabis plays a causal role in the results presented here. The causality of the relationship between cannabis and full-blown psychotic disorders has long been controversial: pleiotropy, reverse causality, bias, and confounding have been proposed as alternative explanations^1,4,34^. Causal inference studies of genetic variants associated with cannabis use and schizophrenia have found reduced^35,36^ or nonsignificant^37^ effects of cannabis use on schizophrenia, while suggesting a reverse causal effect^35,37^. In other words, an alternative explanation is that genetic predisposition to schizophrenia may lead individuals to use cannabis, perhaps due to dysfunction in reward circuitry induced by these genetic factors^38^ or as a means of self-medicating to reduce negative symptoms, anxiety, or insomnia^39–41^.

The use of self-report data for both cannabis use and psychotic experiences brings with it an additional layer of limitations. Self-reporting delusions requires a degree of self-awareness that may be absent in some individuals^42,43^ Some people may be more prone than others to conceal or under-report both cannabis use and psychotic experiences, for instance due to stigma or the illegal status of recreational cannabis in the UK; encouragingly, self-reported cannabis use has been shown to correlate reasonably well with measurements of cannabinoids in the hair and urine in the UK^44^. Although the UK Biobank’s Mental Health Questionnaire asks at what age individuals last used cannabis, it does not ask at what age they first used cannabis and this lack of data leaves open the possibility that some individuals only started using cannabis after their first psychotic experience. Further, it is not clear whether the reported psychotic symptoms occurred during cannabis use or months or years afterwards. The lack of data on recreational use of other drugs implicated in psychosis or psychotic experiences, such as psychedelics (e.g., lysergic acid diethylamide, psilocybin), dissociatives (e.g., ketamine), entactogens (e.g., ecstasy), and stimulants (e.g., methamphetamine), may further confound the results. Participants did not self-report the potency of cannabis consumed^45^ nor its content of cannabidiol, which has antipsychotic properties^46–48^ and (although evidence is mixed^49^) may modulate the putative causal effects of cannabis on psychosis^50^. Also, because cannabis-use frequency was ascertained as the maximum frequency of ever taking cannabis, participants did not report variations in cannabis use frequency over time, which may further complicate interpretation of the results. Finally, the four psychotic experiences ascertained in the UK Biobank Mental Health Questionnaire do not represent the full range of possible positive symptoms—let alone negative symptoms, which participants at clinical high risk for psychosis may in any case be less aware of^51^ and therefore less likely to self-report.

Overall, we find that self-reported cannabis use is dose-dependently associated with self-reported psychotic experience frequency. Cannabis users’ psychotic experiences tend to be earlier-onset and more distressing than non-users, but not more likely to lead to help-seeking. For the first time, we show that genetic predisposition to schizophrenia strongly modulates the association of cannabis with most types of psychotic experiences. At a time when the spread of laws legalizing cannabis for medicinal or recreational use has been accompanied by more relaxed attitudes towards cannabis, our results support the notion that genetics may make some cannabis users more prone to certain psychotic experiences than others, which could enable targeted harm reduction interventions focused on protecting those at the highest risk.
